# Harnessing underutilized food crops for sustainable extruded snack production: a scoping review

**DOI:** 10.3389/fnut.2026.1829365

**Published:** 2026-05-20

**Authors:** Faisal Eudes Sam, Simon Achaglinkame, Francis Kweku Amagloh, Alberta N. A. Aryee

**Affiliations:** 1 College of Enology, Northwest A&F University, Yangling, Shaanxi, China; 2 Department of Food Science and Technology, Faculty of Agriculture, Food and Consumer Sciences, University for Development Studies, Tamale, Ghana; 3 Food Science and Biotechnology Program, Department of Human Ecology, College of Agriculture, Science and Technology, Delaware State University, Dover, DE, United States

**Keywords:** Bambara groundnut, extruded snacks, orange-fleshed sweet potato, quinoa, sesame seed, underutilized crops

## Abstract

Extruded snacks are primarily made from staple cereals. Incorporating underutilized crops can enhance their nutritional value and diversify ingredient supply. Quinoa, Bambara groundnut, orange-fleshed sweet potato (OFSP), and sesame seed are promising candidates, although evidence on their performance in extruded snack products is dispersed across product formats and outcome characterization. This scoping review was conducted to map peer-reviewed studies published from 2000 to 2026 on extruded snack products containing quinoa, Bambara groundnut, OFSP, or sesame seeds. Searches were conducted in Web of Science, Google Scholar, and ResearchGate databases, followed by deduplication and screening of the results. Data charting captured ingredient form and inclusion, extrusion conditions, and outcomes, including expansion and density, hydration indices, texture, color, sensory acceptance, nutrition-related endpoints, antinutritional factors, and shelf-life indicators. Of the 405 records retrieved, 42 studies were included. Quinoa-based snacks were the most frequently studied, and expansion indices, bulk or unit density, water absorption index (WAI), water solubility index (WSI), and instrumental texture were the most often reported. Bambara groundnut studies commonly target protein enrichment and use mixture or response surface designs to relate formulation and process variables to expansion, density, and hydration indices, with limited but informative storage quality data. OFSP studies prioritized provitamin A endpoints alongside expansion, color, texture, and acceptability, with measurable processing losses reported in several product formats. Sesame seed studies emphasized lipid-rich ingredients and reported consistent reductions in expansion with increasing inclusion, while offering stronger coverage of oxidative stability during storage through peroxide and acidity values. Across crops, sensory evaluation, shelf-life, microstructure imaging, antinutritional factors, and protein quality parameters were reported less consistently than physical structure metrics, and energy-based process descriptors were infrequently provided. Research supports the feasibility of these underutilized crops in extruded snack systems; however, translation is constrained by uneven outcome coverage and inconsistent reporting of extrusion severity. Future studies should report processing severity descriptors more consistently and align findings with crop-specific targets, including provitamin A retention for OFSP, oxidation management for sesame seed-enriched snacks, and standardized structure-texture-sensory linkages across formulations.

## Graphical abstract

**Figure:**
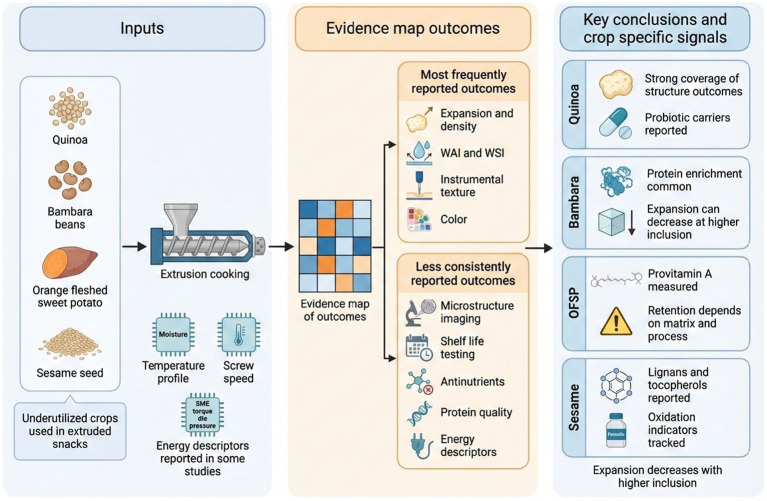

## 1 Introduction

Extrusion is a high-temperature, short-time thermomechanical process in which a hydrated blend is conveyed, mixed, cooked, and shaped under controlled shear and pressure, and then discharged through a die to form products with defined structures (1–3). Extruded products include ready-to-eat snacks, breakfast cereals, pasta, pet foods, and texturized plant protein ingredients. Commercial expanded snack extrudates are commonly produced from starch rich staples such as maize, rice, and wheat because they expand efficiently and deliver crisp textures (3–6). Extrusion remains appealing due to its versatility, high throughput, relatively low cost, energy efficiency, and absence of liquid effluents, which supports large-scale snack manufacturing (1, 3, 7, 8). Product quality attributes, including expansion, cellular structure, density, and water-related functional properties such as water absorption index (WAI), water solubility index (WSI), and water holding capacity (WHC) can be tailored through processing conditions, including barrel temperature, feed moisture, screw speed, and formulation design (4). Texture is critical for consumer acceptance and is strongly influenced by expansion ratio, cell structure, and processing conditions (9).

The global snacks market was estimated at USD 719.18 billion in 2024 and is projected to reach USD 922.08 billion by 2030, reflecting sustained growth in ready-to-eat convenience foods. Extruded snacks alone were estimated at USD 63.38 billion in 2024 and are projected to reach USD 74.52 billion by 2030, representing about 8.8% of the global snacks market by value in 2024 (10, 11). At the same time, consumer and policy pressure increasingly favors healthier snacks that are higher in protein, fiber, and micronutrients and lower in salt, fat, and empty calories (3, 4). As a result, manufacturers are increasingly enriching cereal extrudates with pulses, soy, lentils, chickpeas, barley, and tubers to improve nutritional density while maintaining an acceptable texture (4). Protein enrichment with legumes and other plant ingredients can improve nutrient density, but formulation should also consider digestibility and antinutritional factors that may accompany some plant materials and processing conditions (2, 5). Snacks are also used as delivery vehicles for vitamins, minerals, and bioactive compounds to help address micro- and macro-nutrient deficiencies (9) and to support gluten-free and special diet products using alternative grains and legumes (9).

Conventional extruded snacks are often formulated to optimize cost and texture performance rather than nutrient density (12–14). Consequently, many commercial products are starch-rich and energy-dense, yet relatively low in protein, fiber, micronutrients, and bioactive compounds, and may also rely heavily on wheat, limiting suitability for gluten-intolerant consumers (12, 15). Evidence suggests that cereal-based snacks can be calorie-dense but low in nutritional value (12, 16). Overreliance on a narrow set of staples constrains nutritional diversity, whereas multigrain and cereal-legume extrudates can enhance protein quality, dietary fiber, minerals, vitamins, and antioxidant-related properties such as phenolic content, flavonoid content, and radical scavenging capacity, while remaining sensorially acceptable and, in some cases, showing lower glycemic response indicators (12, 15, 17, 18).

**Figure 1:**
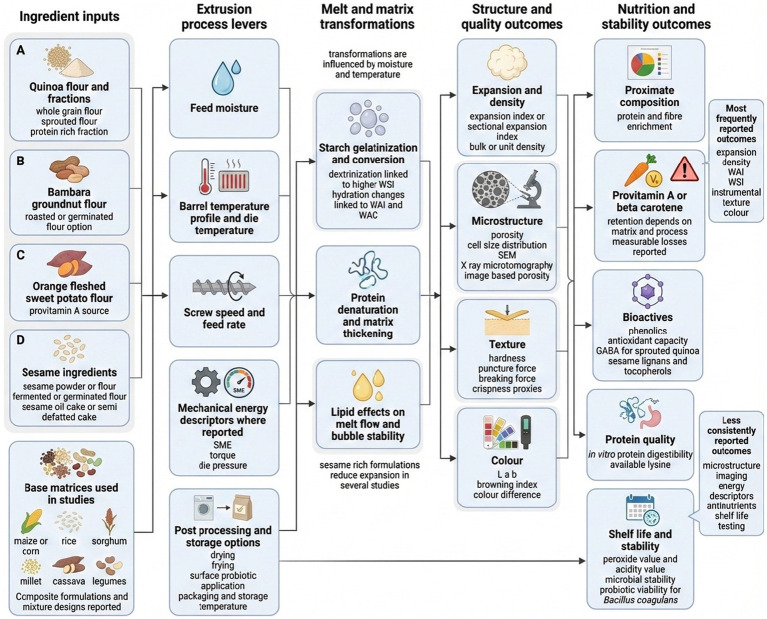
Conceptual framework linking underutilized crop formulation and extrusion processing variables to melt transformation, microstructure formation, texture, sensory attributes, and nutritional and stability outcomes in extruded snack products (15, 23, 25, 28, 31, 36, 38, 43, 46, 48, 52, 56, 58).

Underutilized crops are plant species that are traditionally cultivated in specific regions but have received limited research investment and mainstream adoption, despite their potential to address malnutrition and poverty linked to overdependence on staples such as rice, maize, and wheat (19). In the context of extruded snacks, underutilized crops refers to those that serve as minor ingredients in mainstream industrial snack formulations compared to staple cereals, even when broader cultivation or non-snack uses exist. Underutilized food crops such as quinoa (*Chenopodium quinoa* Wild.), Bambara groundnut (*Vigna subterranea* (L.) Verdc.), orange-fleshed sweet potato (*Ipomoea batatas* L.) (OFSP), and sesame seed (*Sesamum indicum* L.), which offer nutritional benefits and functional properties, may enhance extruded snack formulations. Quinoa provides a complete amino acid profile and is rich in fiber and minerals, including iron, magnesium, phosphorus, and potassium (20). Bambara groundnut is nutrient-dense and drought tolerant, contributing protein, fiber, minerals, and lipids to snack systems (21). OFSP is widely recognized for its provitamin A content and its relevance to vitamin A deficiency interventions (22). Sesame seeds provide healthy fats and essential amino acids, adding both nutritional value and distinctive sensory attributes (23). Despite growing interest, research on extruded snack products incorporating quinoa, Bambara groundnut, OFSP, and sesame seeds remains fragmented and product-specific, with limited crop-focused synthesis of processing behavior, nutritional changes, and application potential (24–29). Therefore, this scoping review aimed to systematically map the existing evidence on the use of quinoa, Bambara groundnut, OFSP, and sesame seeds in extruded snack products. Additionally, key knowledge gaps that may influence future research and product development will be highlighted. It is important to note that the scope was limited to these four crops to ensure consistent charting across divers formulations and to facilitate a more in-depth synthesis of each crop. The conceptual relationships that guided the data charting and synthesis are summarized in Figure 1.

## 2 Methods

### 2.1 Study design

A scoping review approach was used to map the breadth of evidence on extruded snack products formulated with quinoa, Bambara groundnut, OFSP, or sesame seeds and to identify research gaps. Scoping reviews are appropriate when evidence is heterogeneous across product formats, experimental designs, processing conditions, and outcome measurements (30).

### 2.2 Search strategy

A systematic search was conducted using Web of Science, Google Scholar, and ResearchGate to identify studies on extruded snack products incorporating the four target crops. The search covered publications from 2000 to 2026. Truncated keyword combinations were used to capture terms related to crops, extrusion processing, product types, and major outcomes. The core search string combined terms for underutilized or neglected crops and four specific crop names with extrusion-related terms (extrusion, extrusion cooking, extrusion technology, extruded snacks, and snack foods) as well as outcome-related terms (nutritional indicators, proximate composition, provitamin A, functional properties, expansion, bulk density, sensory characteristics, shelf-life, and antinutritional factors).

### 2.3 Selection of sources of evidence

All records were exported, and duplicates were removed using Mendeley Desktop software. Screening proceeded in two stages. First, the titles and abstracts were screened for relevance. Second, potentially eligible studies were included. The final set of included studies was compiled for data charting.

### 2.4 Eligibility criteria

Studies were included if they were peer-reviewed journal articles that reported the development or evaluation of an extruded snack product and contained at least one of the four target crops (quinoa, Bambara groundnut, OFSP, or sesame seeds) in the formulation. The target crop list was defined before screening and was limited to these four crops to ensure consistent charting across heterogeneous formulations and findings. Studies were excluded if extrusion processing was not used, if the work focused on the crop without producing an extruded snack product, or if extruded products were developed from other crops without including at least one of the target crops.

### 2.5 Data charting and synthesis of results

A data charting template was used to extract information on ingredient form and inclusion level, base formulation, extruder type (when reported), key extrusion variables (feed moisture, barrel temperatures, screw speed, and energy-related variables such as specific mechanical energy (SME), if reported), and findings including expansion and density metrics, water absorption index (WAI) and water solubility index (WSI), texture, color, microstructure, sensory evaluation, proximate composition (moisture, protein, fat, ash, carbohydrate, dietary fiber), micronutrients (minerals, vitamins or provitamins), antinutritional factors, and shelf-life indicators. The results were synthesized using descriptive mapping and narrative synthesis organized by crops and by cross-cutting outcome categories.

## 3 Results

### 3.1 Study selection

**Figure 2:**
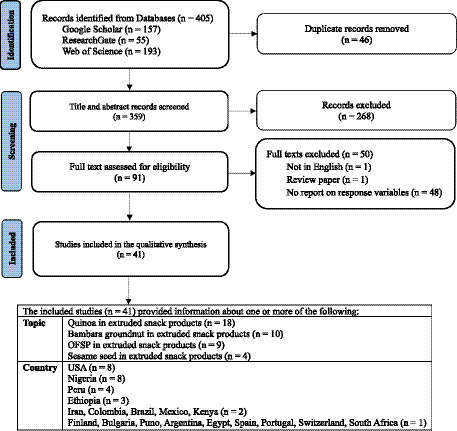
PRISMA flow diagram of study identification, screening, eligibility assessment, and included studies for the scoping review.

A total of 405 records were retrieved (157 from Google Scholar, 55 from ResearchGate, and 193 from Web of Science). After removing 46 duplicates, 359 records were screened by title and abstract, and 268 records were subsequently excluded. Seventy-one full-text articles were assessed for inclusion, and 42 studies were included in the final synthesis. The study selection process is summarized in a PRISMA-style flow diagram (Figure 2).

### 3.2 Characteristics of included studies

The key characteristics of the included studies, such as publication year, country or region, crop category, product format, extruder type, and primary findings, are summarized in Table 1. This overview shows the distribution of evidence across crops and highlights variations in the depth of reporting on extrusion conditions and key findings.

**Table 1:** Characteristics of included studies on extruded snack products formulated with quinoa, Bambara groundnut, orange-fleshed sweet potato, or sesame seed.

Country	Crop	Formulation level (%)	Extrusion type	Processing conditions (temperature/moisture)	Key findings	References
Finland	Quinoa	20–50	Twin screw	140–160 °C/14–18%	Maintained expansion and stiffness; improved folate content	(31)
Peru	Quinoa	1.77	Co-rotating twin screw	40–120 °C/14%	Higher protein content and desirable sensory attributes	(32)
Bulgaria	Quinoa	20	Twin screw	50–150 °C	Increased expansion and reduced bulk density	(33)
Peru	Quinoa	100	Single screw	90–120 °C/12.5–13.5%	Improved WAI, WSI and expansion characteristics	(34)
Iran	Quinoa	25–70	Twin screw	18.9% moisture	Increased antioxidant activity and reduced hardness	(35)
Argentina	Quinoa	14–17	Twin screw	NR	Improved phenolics, GABA and expansion properties	(38)
Egypt	Quinoa	60	Twin screw	100–150 °C/16%	Enhanced protein quality, minerals and sensory acceptability	(44)
Brazil	Quinoa	100	Twin screw	NR	Improved gelatinization and extrusion processability	(37)
Mexico	Quinoa	0–30	Twin screw	NR	Increased nutrient density and sensory quality	(47)
Colombia	Quinoa	10–60	Twin screw	60–110 °C/18%	Improved antioxidants but reduced crispiness at high inclusion	(46)
USA	Quinoa	100	NR	70 °C	Supported probiotic viability and shelf stability	(36)
Brazil	Quinoa	5–20	Single screw	80–120 °C/14–22%	Increased specific volume and solubility	(43)
USA	Bambara groundnut	20–100	Twin screw	100–130 °C	Increased protein and energy; reduced shelf-life at high levels	(28)
USA	Bambara groundnut	20–100	Twin screw	100 °C/27%	Higher inclusion increased microbial load and darker color	(50)
Nigeria	Bambara groundnut	NR	Single screw	NR	Influenced expansion, viscosity and water absorption	(52)
USA	Bambara groundnut	17–80	Single screw	NR	Increased protein and dietary fiber contents	(51)
Nigeria	Bambara groundnut	NR	Single screw	150 °C/20%	Improved sensory properties after processing	(49)
USA	OFSP	10–80	Twin screw	NR	Increased vitamin A but darkened color	(54)
Nigeria	OFSP	50–100	Single screw	100 °C/55%	Protein increased with legume substitution	(63)
Ethiopia	OFSP	35	Twin screw	60–145 °C/21%	Improved energy value but reduced protein	(59)
South Africa	OFSP	20–50	Twin screw	0–80 °C	Affected cooking quality and texture	(55)
Nigeria	OFSP	17–100	Single screw	105 °C	Increased *β*-carotene but reduced expansion	(61)
Kenya	OFSP	50–100	Twin screw	90 °C/35%	Produced nutrient-dense complementary foods	(58)
Ethiopia	OFSP	10	Twin screw	120 °C/22%	High carbohydrate contribution	(60)
Iran	Sesame seed	5–15	Twin screw	50–140 °C	Increased phenolics but reduced expansion	(23)
Brazil	Sesame seed	0–20	Single screw	60 °C/5%	Improved protein and sensory quality	(56)
Nigeria	Sesame seed	15–30	Single screw	NR	Improved nutrient composition and acceptability	(24)
Mexico	Sesame seed	5–30	Single screw	139 °C/23%	Optimal acceptance at 10% inclusion	(57)

NR = not reported.

### 3.3 Quinoa in extruded snack products

Quinoa-based extruded snack products were the most frequently studied among the four target crops included in this scoping review. However, the reported outcomes reveal distinct formulation-dependent challenges that can be interpreted using established concepts of extrusion structure formation. Studies evaluated quinoa in directly expanded cereal-based snacks, quinoa-rich gluten-free extrudates, and second-generation snack systems (snacks produced from reprocessed extrudate materials such as pellets that are expanded in a separate step by baking or frying), with quinoa incorporated as whole grain flour, sprouted flour, and refined or protein-enriched fractions depending on the formulation objective (31–48). Table 2 summarizes ingredient forms and inclusion levels, extrusion conditions reported, and key findings measured.

**Table 2:** Summary of quinoa based extruded snack studies: formulation characteristics, extrusion conditions, and reported outcomes.

Country	Formulation (quinoa; %)	Extrusion type	Processing conditions (temperature/moisture)	Findings	References
Finland	20–50	Twin screw	140–160 °C/14–18%	This study showed that it is possible to produce maize-based extrudates at various contents of amaranth or quinoa, ranging from 20 to 50% of solids, without any substantial reduction in sectional expansion or increase in stiffness Extrudates containing quinoa presented the highest content of folate Extrudates containing 20% quinoa had a slightly lower content of phenolic compounds compared to those containing 20% amaranth	(31)
Peru	1.77	Co-rotating twin screw	40, 47, 56, 77, 97, 112, and 120 °C/14%	It was observed that the ME has a lower SEI (2.74 g/cm^3^) compared to the commercial CE and WE (*p* < 0.05). WSI values were lower in the ME compared to the CE and the WE samples (*p* < 0.05) The BD was slightly higher (0.27 g/cm^3^) The protein content of the ME was 13.6%, which was significantly higher than the commercial extrudates (*p* < 0.05) Meanwhile, the lipid content of the ME was lower than the QE (*p* < 0.05) Regarding crude fiber, the ME had 2.20%, which was lower than the QE but higher than the CE and the WE According to the flash sensory profiling method, the ME was found to be closely related to the chocolate flavor attribute, brown color, and crunchiness. In addition, the free ranking task method described the ME as an extruded product with a natural appearance and chocolate taste	(32)
Bulgaria	20	Conical, counter-rotating, twin-screw	50, 80, 100, 150 °C	The addition of quinoa showed higher expansion index and porosity as compared to plain maize The addition of quinoa to the formulations yielded the extrudates with the lower bulk density (0.046 ± 0.001 g/cm^3^)	(33)
Puno, Peru	100	Single screw	90–120 °C/12.5 and 13.5%	The moisture content of the extrudates ranged between 3.08 and 6.12%, while firmness values varied from 7.25 N to 25.86 N, showing the significant influence of extrusion temperature on texture. The water solubility index (WSI) displayed a wide range, from 0.17 to 71.61%, suggesting high starch dextrinization during extrusion. The WAI increased notably, indicating extrusion-induced physical changes. Additionally, the sectional expansion index (SEI) ranged from 7.33 to 13.08, reflecting the impact of extrusion on the final product’s structure	(34)
Iran	25, 50, and 70	Co-rotating twin screw	18.94%	Increased quinoa inclusion in the extrudates increased the porosity, WAI, and antioxidant properties, along with reduced hardness. The extruded flour exhibited improved particle size, protein, total dietary fiber, soluble fiber, antioxidant activity, and functional properties. Notable improvements in flowability (Carr index and Hausner ratio), dispersibility, a* and b* attributes were observed in the extruded flour	(35)
Argentina	14 and 17	Twin screw	NS	Partial incorporation of sprouted quinoa flour (SQF) and cañihua flour (SCF) in maize grits (CG)-based extrudates increased phytic acid (PA), total soluble phenolic compounds (TSPC), *γ*-aminobutyric acid (GABA) and oxygen radical antioxidant activity (ORAC) of the extrudates. Sprouted grain flour usually results in a deleterious effect physicochemical properties of extrudates, but the partial mixture of CG with SQF and SCF circumvented the negative effect of germinated flours, improving technological properties, favoring the expansion index and bulk density and increasing water solubility	(38)
USA	60–75	Single screw		The results indicate that for the formulations obtained, the lower the content of quinoa flour, the higher the value of protein	(39)
Egypt	60	Co-rotating twin-screw	100 °C, 130 °C and 150 °C/16%	Quinoa provides superior nutritional qualities to the extrudates, including high-quality protein with balanced amino acids (high lysine and methionine), increased minerals (iron, phosphorus, calcium), B vitamins, and dietary fiber compared to traditional cereals. Quinoa-based extrudates with blends containing at least 15% rice (e.g., Quinoa:Maize:Rice at 60:25:15 and Quinoa:Oats:Rice at 60:25:15) showed higher expansion ratio, lower bulk density, lighter color (higher L*), and better sensory acceptability	(44)
Colombia	30–65%	Twin screw	75, 81, 90, 80, 95, 70, 75 °C/30%	Higher quinoa flour resulted in higher pasting temperature. Quinoa flour reduced the final viscosity. A positive correlation was observed between defatted hyper-protein quinoa flour (HDQF) and viscosity, and a negative correlation with non-defatted hyper-protein quinoa flour (HQF). Regarding thermal properties, it was found that all blends showed low gelatinization enthalpy values, attributed to the high proportions of HQF and HDQF)	(41)
Brazil	100	Twin screw	NS	Quinoa’s relatively high fat content (7.2%) contributed to lower SME values during extrusion (170–402 kJ/kg), which were lower compared to other cereals. The fat acts as a lubricant, reducing melt viscosity and shear stress in the extruder, thus affecting the extent of starch gelatinization and expansion Quinoa extrusion under optimized conditions (low moisture, low temperature, medium screw speed) resulted in extrudates with desirable physicochemical properties, including maximum expansion, minimum density, high degree of gelatinization, and controlled water solubility, confirming quinoa’s good processability and potential as a novel extrusion raw material	(37)
Mexico	0, 10, 20, and 30	Twin screw	NS	The addition of quinoa significantly improved the protein, lipid, dietary fiber, and mineral content of extruded flips. Products containing 30% quinoa showed the highest nutrient density. Quinoa enrichment also positively influenced protein quality by enhancing both essential and non-essential amino acid profiles. The incorporation of quinoa favorably modified the fatty acid composition of the extrudates, increasing *α*-linolenic acid content while maintaining desirable MUFA and PUFA profiles. Sensory evaluation indicated that quinoa enrichment, particularly at the 20% level, enhanced key sensory attributes, while higher screw speeds improved crispiness and expansion without negatively affecting flavor. Response surface methodology confirmed that quinoa level was the dominant factor influencing nutritional and quality attributes, whereas screw speed mainly contributed to physical structure and sensory texture	(47)
Colombia	60, 30, 20, and 10	Twin screw	60 °C, 80 °C, and 110 °C/18%	The crispiness of the extruded foods changed with the addition of quinoa. It was found that the higher the amount of quinoa flour, the lower the crispiness The addition of quinoa improved the antioxidant properties and bound phenolic compounds The 60% inclusion had the highest rating for color, and the lowest for texture, aroma and flavor	(46)
USA	100		70 °C	The results of the study showed that the quinoa probiotic snacks made with the addition of *B. coagulans* GBI-30 have a final concentration of approximately 107 UFC/g, and can be stored for 120 days at room temperature, low humidity, and aw: 0.2 ± 0.2 without affecting their quality	(25)
Peru	NS	Single screw	NS	Quinoa blends had the lowest expansion ratios of 0.92 and 0.99 Quinoa blends had the lowest pellet durability index (PDI) As the quinoa particle size decreased, the a* and b* values decreased in the extrudates	(45)
Spain	13	Pastaia 2 extruder	NS	The firmness increases as the concentration of *cushuro* flour decreases and decreases as the concentration of sprouted quinoa flour increases	(42)
Portugal	20, 35, and 50	Twin screw	NS	Extrudates containing amaranth and quinoa were rated as crispier. There was a strong perception of hard particles in extrudates containing amaranth and quinoa The incorporation of up to 35% quinoa increased SEI and had a minor effect on stiffness; further addition of quinoa (up to 50%) decreased SEI and increased stiffness Extrudates containing 20% quinoa had around 77% of their pore volume between 0 and 2000 mm, whereas those containing 50% quinoa had around 80% There was a considerable increase in wall thickness as the content of quinoa increased to 50%	(62)
Switzerland	100	Double screw	40, 80, 120, and 140 °C	Lesser changes in WSI levels were shown for quinoa (12.4%) and sorghum (9.2%) Extruded sorghum, quinoa, millet and teff had similar sectional expansion index (SEI) (14, 13, 12 and 11, respectively)	(36)
Brazil	5, 10, 15, and 20	Single screw	80, 90, 100, 110, and 120 °C/14, 16, 18, 20 and 22%	Higher levels of quinoa flour increased the specific volume of the extruded products Higher levels of water solubility are observed in conditions of high amount of quinoa flour in the mixture and low screw speed, as well as, under conditions of high temperature and high amount of quinoa flour Snacks with good technological characteristics can be obtained under the conditions of 10% of quinoa flour, 250 rpm of screw speed, temperature of 100 °C, and moisture of 15%	(43)

NS = not stated.

Across quinoa studies, the most commonly reported findings often centered on water-related functional indices (WAI and WSI) and structure-forming attributes, particularly expansion metrics, density, and porosity (34, 36, 43). In an extruded quinoa snack model, changes in WSI and WAI were discussed in relation to extrusion induced starch transformations, including dextrinization, during combined thermal and mechanical treatment (34). Process variables most frequently examined included feed moisture, barrel or die temperature, and screw speed, with several studies also reporting processing severity descriptors such as SME, torque, or pressure (31, 36). Feed moisture influences melt viscosity and the extent of mechanical energy dissipation, which can consequently alter expansion and density by affecting bubble growth and setting. The barrel temperature profile and screw speed interact with shear and residence time to impact starch conversion and fragmentation, observable in trends of WAI and WSI as well as in textural outcomes (31, 34, 36). These response and process variables provide a common basis for comparing how quinoa modifies melt behavior and final structure across formulations, although comparability is often reduced by incomplete reporting of extrusion settings and variations in measurement protocols (36, 43, 44).

Comparisons across studies show that processing variables consistently influence quinoa performance within each matrix. However, the net effect of quinoa inclusion on expansion is not uniform across formulations. Under certain conditions, adding quinoa to maize-based extrudates reduced bulk density while increasing expansion and porosity, with microscopy revealing a more expanded cellular structure consistent with lower-density products (33). Conversely, in quinoa-rich and protein-enriched formulations, reduced expansion and increased hardness have been reported, aligning with a shift toward denser structures when protein and fiber fractions increase relative to starch or when processing severity and moisture conditions limit gas cell growth and stabilization (32, 46). This discrepancy is best understood as a matrix composition effect rather than a contradiction, as quinoa may enhance expansion in starch-rich cereal bases, while a high inclusion of protein-rich quinoa fractions can increase melt viscosity and restrict bubble growth, resulting in lower expansion and higher hardness (32, 33, 46).

Studies that reported processing severity descriptors enhance cross-study interpretation because set points alone do not define mechanical energy dissipation. In quinoa-based extrusion research, feed moisture, thermal settings, and screw speed were examined alongside variables such as SME, torque, and die pressure in a subset of studies, supporting the conclusion that moisture and temperature modulate both mechanical energy input and starch conversion, thereby influencing expansion and textural hardness (31, 36, 37). When moisture is higher, melt plasticization can reduce mechanical energy dissipation and molecular fragmentation, which can shift WSI and reduce expansion depending on the formulation and die conditions. Conversely, when moisture is lower or shear is higher, increased fragmentation can enhance WSI and alter cell wall formation, which may increase crispness in some systems but also lead to greater hardness when expansion is reduced (36, 37, 43). These trends are reflected in the reported co-variation of WSI, expansion, and hardness across quinoa extrudate datasets (34, 37, 43).

Microstructure and sensory assessments provide further evidence that quinoa-driven changes in expansion result in perceivable texture differences. In maize-based extrudates containing quinoa, sensory descriptors such as crispness, crunchiness, hardness, and adhesiveness were linked to pore structure quantified by X-ray microtomography, highlighting the role of cellular architecture and wall thickness distribution in shaping consumer-perceived texture (48). Quinoa studies that quantified porosity through image analysis and assessed fracture-related texture similarly support the value of combining structural measures with sensory data for interpretation beyond expansion indices alone (46). Such integration remains limited across the broader quinoa literature. However, where applied, it strengthens the ability to relate process and formulation changes to textural drivers of acceptance (44, 46, 48).

Pretreatment studies indicate that quinoa functionality can be optimized before extrusion, affecting hydration indices and bioactive-related outcomes. Sprout-based formulations altered WAI and WSI patterns, consistent with germination-driven compositional and enzymatic shifts that change the balance between soluble fragments and structure-forming components after extrusion (35, 36, 38). Functional snack concepts also extend quinoa extrusion toward delivery applications. *Bacillus coagulans* fortified quinoa snacks tested both pre-extrusion inclusion and post-extrusion surface application approaches, quantifying viability during storage and resistance in simulated gastrointestinal conditions. This demonstrates that quinoa extrudates can be engineered as probiotic carriers while still achieving the standard structure and texture outcomes used to evaluate snack quality (25, 46). Overall, the quinoa evidence base supports feasibility across product formats. However, mechanistic interpretation and cross-study comparability would be enhanced by more consistent reporting of extrusion severity descriptors and more frequent pairing of microstructure and sensory measurements with expansion and hydration indices.

### 3.4 Bambara groundnut in extruded snack products

The studies on Bambara groundnut-based extruded snacks, formulation strategies, and reported outcomes are summarized in Table 3. Across several studies, Bambara groundnut was primarily incorporated as flour in cereal and root-based matrices to enhance protein density and broaden the nutritional profile of extruded snacks. Product performance was commonly evaluated using metrics such as expansion ratio, bulk density, texture or breaking force, WAI, WSI, color, proximate composition, and sensory acceptance (49–52).

**Table 3:** Summary of Bambara groundnut based extruded snack studies, formulation characteristics, extrusion conditions, and reported outcomes.

Country	Formulation (Bambara groundnut; %)	Extrusion type	Processing conditions (temperature/moisture)	Findings	References
USA	20, 40, 60, 80, and 100	Co-rotating twin screw	100 and 130 °C	Protein (4.08–15.03 g/100 g), fat (4.20–12.74 g/100 g), fiber (5.29–6.46 g/100 g), ash (0.09–4.80 g/100 g), and energy (366.13–396.9 kcal/100 g) increased with increasing proportion of Bambara groundnut in the formulation The provitamin A content in OFSP-Bambara groundnut blend decreased with increasing Bambara groundnut levels Snacks made from 100% Bambara groundnut flour were liked the list but addition of OFSP significantly improved their acceptability scores Estimated shelf-life decreased with increasing proportion of Bambara groundnut in the formulation	(28)
USA	100, 80, 70, 60, 50, 40, 30, and 20	Twin screw	100 °C/27%	The bacterial load diminishes as the proportion of Bambara groundnut in the extrudates formulation reduces. The control sample, M1 (extruded samples made from 100% Bambara groundnut flour), exhibited the highest total bacterial counts varying from 1.48 to 6.84 CFU/g and 1.48 to 7.11 cfu/g Extrudates with high Bambara substitution was observed to be dark at month 0 An increase in texture values was noted in the puffed samples as the proportion of Bambara groundnut in the flour blend formulation decreased	(50)
Nigeria	NS	Single screw	NS	The result shows that feed moisture and level of Bambara groundnut both influenced the expansion ratio *Fura* extrudates shows that increasing feed moisture significantly decreased the WAI of extrudate, while increase in the level of Bambara groundnut flour resulted in increased WAI. Increasing screw speed and level of Bambara groundnut flour were found to increase the WSI. The extrudates showed that increase in the amount of Bambara groundnut flour increased apparent viscosity of extrudate	(52)
USA	80, 10, 50, 33, 67, and 17	Single screw	NS	Bambara ground nut and cassava starch inclusion significantly had a positive interactive effect on carbohydrate and a negative interactive effect on total dietary fiber The protein content ranged from 3.26 to 17.62%. Protein content increased and was highest at high level of Bambara groundnut inclusion The crude fiber and total dietary fiber contents of the snacks ranged from 0.32 to 4.78% and 7.36 to 28.74%, respectively. The increased crude fiber and total dietary fiber contents with increase in the levels of Bambara groundnut flour and maize bran and decrease with increase in cassava starch level was due to the high fiber content of maize bran and Bambara groundnut flour	(51)
Nigeria	NS	Single screw	150 °C/20%	Extruded snacks made with raw and processed Bambara groundnut flour samples differed significantly in terms of color, taste, flavor and overall acceptability. However, the extrudates did not differ significantly in their texture and mouthfeel	(49)
Nigeria	17, 33, 50, 66, and 100	Single screw	105 °C	There was a decrease in expansion ratio (ER) with the inclusion of OFSP extrudate, while the ER increased with the inclusion of African breadfruit extrudate Hard ness of the thermo-extrudate may be attributed to the lower expansion ratio and high bulk density at high OFSP inclusion The *β*-carotene for the extrudates ranged from 0.004 to 13.62 mg/100 g. Significant (*p* < 0.05) differences existed in all the parameters measured. Results showed that there was a significant increase in β-carotene value with increase in OFSP flour	(61)
Kenya	100, 50, and 54	Twin screw	90 °C/35%	Compositing soybean and amaranth seeds with OFSP make the extrudates a valuable source of nutrients and may be utilized in the preparation of many foods, particularly as fundamental components in enhancing other foods such as infant and baby foods and any other foods.	(58)
USA	100, 80, 60, 40, and 20	Co-rotating twin screw	100 and 130 °C	Moisture (4.79–8.34 g/100 g), carbohydrate (55.53–78.99 g/100 g), and provitamin (0.54–17.33 mg/100 g) contents of the snacks increased with increase in the proportion of OFSP in the formulation Snacks made from 100% Bambara groundnut flour were liked the list but addition of OFSP significantly improved their acceptability scores	(28)
Nigeria	14.71	Twin screw	100 and 120 °C	Higher temperatures enhance porosity and overall acceptability in the OFSP- enriched snack	(53)
Ethiopia	10	Twin screw	120 °C/22%	The lowest value of protein was that of OFSP (5.70 g/100 g). Crude fat also recorded lowest for OFSP (0.84 g/100 g). Crude fiber contents ranged from 3.76 for OFSP to 5.76 g/100 g. The OFSP gave the highest value of carbohydrate (86.09 g/100 g)	(60)

The inclusion of Bambara groundnut was linked not only to nutritional enrichment but also to structural and texture trade-offs that depended on the complementary ingredients and processing parameters. In blends designed for puffed products, response surface and mixture design approaches demonstrate that Bambara groundnut levels, feed moisture, and screw speed jointly influence expansion ratio, bulk density, WAI, and WSI, with higher moisture and Bambara groundnut levels generally reducing expansion and shifting hydration indices in several formulations (51, 52). In co-extruded snacks containing OFSP and Bambara groundnut, increasing the proportion of Bambara groundnut raised the levels of protein, fat, crude fiber, ash, and energy, while a higher proportion of OFSP increased provitamin A content (28). In a separate Bambara groundnut snack formulation where the blend ratio was fixed and the Bambara seeds were pretreated before milling, roasting and germination altered proximate composition, functional properties, and antinutrient levels in the resulting extrudates. Roasted and germinated Bambara flours exhibited higher protein content than untreated and germinated samples, and antinutrient activities varied across treatments, with trypsin inhibitor activity reduced by 37.5% after roasting, by 17% after germination, and by 68% when germination was followed by roasting. The pretreatments also resulted in significant differences in water binding capacity, foaming capacity, and nitrogen solubility, with snacks made from roasted Bambara flour receiving the highest scores for taste, aroma, flavor, and overall acceptability (49).

Shelf-life and storage stability have been more directly addressed in Bambara groundnut-based extrudates compared to several other underutilized crop categories, largely due to concerns regarding lipid oxidation and microbial stability. In OFSP and Bambara groundnut snacks, peroxide value tracking indicated a shorter predicted shelf-life as Bambara groundnut levels increased, consistent with greater susceptibility to oxidative changes in higher Bambara groundnut formulations (28). Storage studies of puffed snacks made from pearl millet and Bambara groundnut further demonstrate that temperature and storage duration influence moisture gain, microbial counts, and sensory scores, with more rapid quality loss occurring at higher storage temperatures, underscoring the need for packaging and storage control when Bambara groundnut is used in expanded snack formats (50). The literature on Bambara groundnut snacks consistently addresses parameters such as expansion and density, hydration indices, texture, and proximate composition. However, comparability across studies is constrained by differences in base matrices used, inconsistent reporting of extrusion conditions, and variable focus on antinutrient content and shelf-life characteristics among different formulations (28, 49–52).

### 3.5 OFSP in extruded snack products

Studies incorporating OFSP into extruded snacks are summarized in Table 4, including outcomes related to provitamin A or *β*-carotene. The literature indicates that OFSP has primarily been used as flour in cereal- and legume-based formulations to enhance provitamin A content while preserving the expanded structure and crisp texture expected in snack products (15, 28, 53, 54). Table 4 details the ingredient form and inclusion level, reported extrusion settings, and the physicochemical, sensory, and nutrition-related endpoints used to evaluate OFSP-enriched extrudates.

**Table 4:** Summary of OFSP based extruded snack studies, formulation characteristics, extrusion conditions, and reported outcomes.

Country	Formulation (OFSP; %)	Extrusion type	Processing conditions (temperature/moisture)	Findings	References
USA	10, 20, 30, 40, 50, 60, 70, and 80	Twin screw	NS	The L∗ and ΔE values in the extruded samples decreased with a higher proportion of OFSP in the flour blends. Increasing the substitution of sorghum flour with OFSP flour caused the darkening of the extruded snacks, as shown by the declining L∗ and ΔE and increasing a∗ values The levels of titratable acidity in the extruded samples demonstrated an increase corresponding to the rise in the percentage of OFSP flour used in the production of the puffed snack There was a decrease in the moisture content as the proportion of OFSP flour increased in the puffed snack formulation. The moisture content reduced from 5.67% in the S_100_ (100% sorghum) samples to 3.94% in the S_20_O_80_ (samples composed of 20% sorghum and 80% OFSP flour blends) puffed snacks Consistent with the moisture content pattern, there was a decrease in the fat, protein, and crude fiber content as the proportion of OFSP flour increased in the puffed snack formulation. In contrast, the level of carbohydrates in the extruded samples demonstrated an increase corresponding to the rise in the percentage of OFSP flour used in the production of the puffed snack As the proportion of OFSP substitution rose in the puffed snacks, there was a corresponding increase in vitamin A, B1, and C levels. The values rose from 0.24, 0.15, and 0.21 mg/100 g in sample S_100_ to 1.30, 0.19, and 1.82 mg/100 g in the S_20_O_80_ puffed snack, respectively The inclusion of OFSP in samples S_20_O_80_ and S_70_O_30_ resulted in their low appearance and color scores due to the reddish color imparted by OFSP on the extrudates	(54)
Nigeria	100, 90, 80, 70, 60, and 50	Single screw	100 °C/55%	There was significant (*p* < 0.05) difference among the blends for moisture content with 100% OFSPF1 having the highest value while OFSPF with 50% pigeon pea flour substitution had the least value for both varieties The protein, fat and crude fiber contents of both the flour blends and extrudates increased as pigeon pea flour (PPF) increased for the two varieties of OFSP	(63)
Ethiopia	35	Twin screw	60 °C (for zone-6), 60 °C (for zone-5), 80 °C (for zone-4), 100 °C (for zone-3), 120 °C (for zone-2), and 145 °C (for zone-1)/21%	Blends with higher OFSP content tend to have lower protein levels since OFSP is low in crude protein compared to brown teff and dark red kidney beans. The study found that some extruded blends with relatively high OFSP content exhibited a bitter aftertaste, likely due to the dry heat causing formation of a bitter compound known as ipomeamarone in OFSP. This bitterness affected sensory acceptance, with blends high in OFSP less preferred in terms of aftertaste and overall acceptability Extrusion processing with OFSP-containing blends resulted in significantly reduced moisture content compared to raw blends, which helps inhibit microbial growth and prolongs shelf-life OFSP being high in carbohydrates increases the carbohydrate and energy contents of the blends; carbohydrate content ranged 67.10–76.29% in raw flours, and extruded blends had carbohydrates from 72.66–83.96% with energy values around 343.07–356.74 Kcal/100 g, supporting the energy requirement for infants	(59)
South Africa	20, 30, and 50	Twin screw	0, 70, 80, 80, and 80 °C for zones 1 through 5, respectively.	The extruded pasta strands with higher OFSP content tend to have smaller diameters (e.g., from 1.4 mm for 100% maize to 1.1 mm for 50% maize: 50% OFSP), which affects water penetration during cooking and texture attributes. The progressive increase in the proportion of OFSP flour affects the cooking quality and the nutritional properties of the pasta	(55)
Nigeria	17, 33, 50, 66, and 100	Single screw	105 °C	There was a decrease in expansion ratio (ER) with the inclusion of OFSP extrudate, while the ER increased with the inclusion of African breadfruit extrudate Hard ness of the thermo-extrudate may be attributed to the lower expansion ratio and high bulk density at high OFSP inclusion The β-carotene for the extrudates ranged from 0.004 to 13.62 mg/100 g. Significant (*p* < 0.05) differences existed in all the parameters measured. Results showed that there was a significant increase in β-carotene value with increase in OFSP flour	(61)
Kenya	100, 50, and 54	Twin screw	90 °C/35%	Compositing soybean and amaranth seeds with OFSP make the extrudates a valuable source of nutrients and may be utilized in the preparation of many foods, particularly as fundamental components in enhancing other foods such as infant and baby foods and any other foods	(58)
USA	100, 80, 60, 40, and 20	Co-rotating twin screw	100 and 130 °C	Moisture (4.79–8.34 g/100 g), carbohydrate (55.53–78.99 g/100 g), and provitamin (0.54–17.33 mg/100 g) contents of the snacks increased with increase in the proportion of OFSP in the formulation Snacks made from 100% Bambara groundnut flour were liked the list but addition of OFSP significantly improved their acceptability scores	(28)
Nigeria	14.71	Twin screw	100 and 120 °C	Higher temperatures enhance porosity and overall acceptability in the OFSP- enriched snack	(53)
Ethiopia	10	Twin screw	120 °C /22%	The lowest value of protein was that of OFSP (5.70 g/100 g). Crude fat also recorded lowest for OFSP (0.84 g/ 100 g). Crude fiber contents ranged from 3.76 for OFSP to 5.76 g/100 g. The OFSP gave the highest value of carbohydrate (86.09 g/100 g)	(60)

NS = not stated.

Findings for the OFSP snacks included expansion ratio or related indices, bulk or apparent density, instrumental color, and texture metrics, along with proximate composition and micronutrient endpoints associated with provitamin A (15, 53, 54). The effects of formulation were generally product-dependent. In sorghum-based snacks, increasing the proportion of OFSP elevated vitamin A content and altered color (with lightness, L*, and color change, ΔE values decreasing, leading to the darkening of the extruded snacks), while reducing protein and fiber; overall acceptability remained highest for formulations with a greater sorghum fraction (54). In composite puffed snacks designed for nutrient enrichment, mixture design approaches blending OFSP with maize, beans, and amaranth reported improvements in nutrient density and protein digestibility, with favorable consumer acceptance testing among adults and children (15). A process optimization study further revealed that feed moisture and extrusion temperature interact with OFSP levels to influence expansion and density, with reported reductions in expansion ratio and porosity alongside increased density at the specified operating parameters (53).

Retention of provitamin A is a critical outcome in OFSP extrusion studies. In snacks coextruded from OFSP and Bambara groundnut, processing led to losses of provitamin A, with retention levels varying based on blend ratios (higher OFSP inclusion) and extrusion conditions such as temperature and screw speed (28). Evidence from other extruded OFSP products also supports the conclusion that *β*-carotene can decrease during extrusion processing, indicating susceptibility to thermal and oxidative degradation, although the extent depends on the matrix and processing severity (55). Shelf-life has been assessed in OFSP-based extruded snacks through lipid oxidation indicators. In the OFSP and Bambara groundnut system, peroxide value-based modeling indicated predicted shelf-life differences across formulations, with shorter predicted shelf-life at higher Bambara groundnut proportions, consistent with increased lipid content and oxidative risk (28).

### 3.6 Sesame seed in extruded snack products

Sesame seeds present a distinct formulation challenge in expanded snacks due to their oil, protein, and minor bioactive compounds, which can alter melt behavior while also contributing to flavor and oxidative stability. Sesame seeds have been incorporated into snack matrices in various forms, including whole sesame seeds, powder or flour, fermented or germinated sesame seed flour, and sesame seed oil cake or semi-defatted cake used as a protein-rich coproduct ingredient, most often blended with cereal- or cassava-based matrices (23, 24, 56). Studies on sesame seed-based snacks, including extrusion conditions, are summarized in Table 5, which outlines the primary endpoints used to evaluate structure, texture, and storage stability.

**Table 5:** Summary of sesame seed based extruded snack studies, formulation characteristics, extrusion conditions, and reported outcomes.

Country	Formulation (sesame seed; %)	Extrusion type	Processing conditions (temperature/moisture)	Findings	References
Iran	5, 10 and 15	Twin screw	50, 100, and 140 °C,	Addition of sesame powder to puffed maize snack materials caused significant changes (*p* < 0.05) in the fatty acid profile Because of the high level of oleic acid in sesame seed, increasing the percentage of sesame powder in the formulation leads to a significant increase (*p* < 0.05) in oleic acid content in the processed samples Increasing the contents of sesame powder, which contains a high level of phenolic compounds, showed a significant increase (*p* < 0.05) in the total phenolic compounds (TPC) of the formulated samples Expansion ratio of extrudates with 0–15% sesame powder varied from 21 to 45 mL in a 5-g sample. Addition of sesame powder significantly reduced (*p* < 0.05) the expansion ratio of the snacks The effect of sesame powder level on the overall acceptability of puffed maize snack indicated that samples with 5 and 10% had the good acceptance, but higher amounts of sesame powder in the formulation led to a reduction in product acceptability	(23)
Brazil	0 to 20	Single screw	60 °C/5%	The addition of sesame increased protein, fat, and ash content of maize extrudates, whereas carbohydrate content was reduced. The addition of sesame reduced the sectional expansion of the maize extrudates and increased the puncture force. Sesame–maize extrudates were darker than non-sesame-maize extrudates. Increasing the amount of sesame increased the number of cells similar to those of commercial maize extrudates with small cells. Sensory analysis showed 20% sesame-maize extrudates to be acceptable and nutritionally balanced. The use of sesame on maize extrudates up to 20% is an alternative to improve nutritional value, keeping good sensory characteristics	(56)
Nigeria	15 and 30	Single screw	NS	This study has shown that extruded snacks produced from yellow cassava flour substituted with processed sesame seed flour are nutritionally endowed with reduced anti-nutritional components. Processed sesame seed inclusion in cassava flour did not have any negative effect on the products but rather improved the nutrient contents and sensory attributes of the products. Both flours and extrudates produced with 30% inclusion of fermented sesame seeds flours to yellow cassava flour were the highest in terms of functionality, nutrient and acceptability	(24)
Mexico	5, 10, 15, 20, 25 and 30	Single screw	139 °C/23%	The beverage made with 10% extruded sesame seed flour showed the highest global acceptance by the panelists A decrease in acceptance was observed as the concentration of sesame seed flour and the intensity of the beverage color increased. The smell of the beverages was more accepted when the concentration of extruded sesame seed flour was increased	(57)

Sesame seed-enriched extrudates typically include expansion indices, bulk density, and related structural metrics such as porosity, cell size, wall thickness, and specific volume, along with instrumental color and texture metrics like puncture force and fracture behavior, reflecting the sensitivity of puffing and crispness to lipid and protein-rich additions (23, 56). In maize-based expanded extrudates, the inclusion of sesame seed powder reduced the expansion ratio as the sesame seed level increased, while enhancing nutritional and phytochemical composition through higher levels of phenolics and sesame seed lignans, as well as increasing gamma tocopherol, sesamin, and sesamolin in the finished product (23). Similar structure and texture trade-offs were observed in maize extrudates formulated with semi-defatted sesame seed cake. Increasing sesame seed cake reduced the sectional expansion index and increased puncture force while also darkening the extrudates. Microstructure observations via scanning electron microscopy (SEM) supported changes in cell morphology consistent with the measured textural response (56).

Storage stability in sesame seed-enriched extruded snacks has been explicitly addressed through oxidation indices. In puffed maize snacks fortified with sesame seed powder, peroxide value and acidity were monitored during storage, supporting the use of sesame seed-enriched formulations in oxidative stability assessments alongside sensory evaluations and suggesting an optimal inclusion range within that product system (23). In cassava-based snacks produced with processed sesame seed flours, pretreatments such as fermentation and germination were evaluated. The resulting snacks showed improvements in proximate composition and reduced antinutrient levels compared with the flour blends, with sensory scores indicating high acceptability at higher levels of fermented sesame seed inclusion (24). Additionally, there is evidence that extrusion can modify the phenolic profiles and *in vitro* bioactivity of sesame seed byproduct ingredients in non-snack applications, which may be relevant when considering sesame seed coproducts as functional ingredients, although such findings should be interpreted separately from expanded snack structure outcomes (57). Based on the literature, the inclusion of sesame seeds offers a clear pathway to enhance extruded snacks with lipid-associated bioactive compounds and flavor attributes, while consistent reductions in expansion at higher inclusion levels highlight the need for formulation and processing optimization to maintain texture and structure in expanded snack formats (23, 56).

### 3.7 Cross-cutting evidence map and reporting gaps

**Figure 3:**
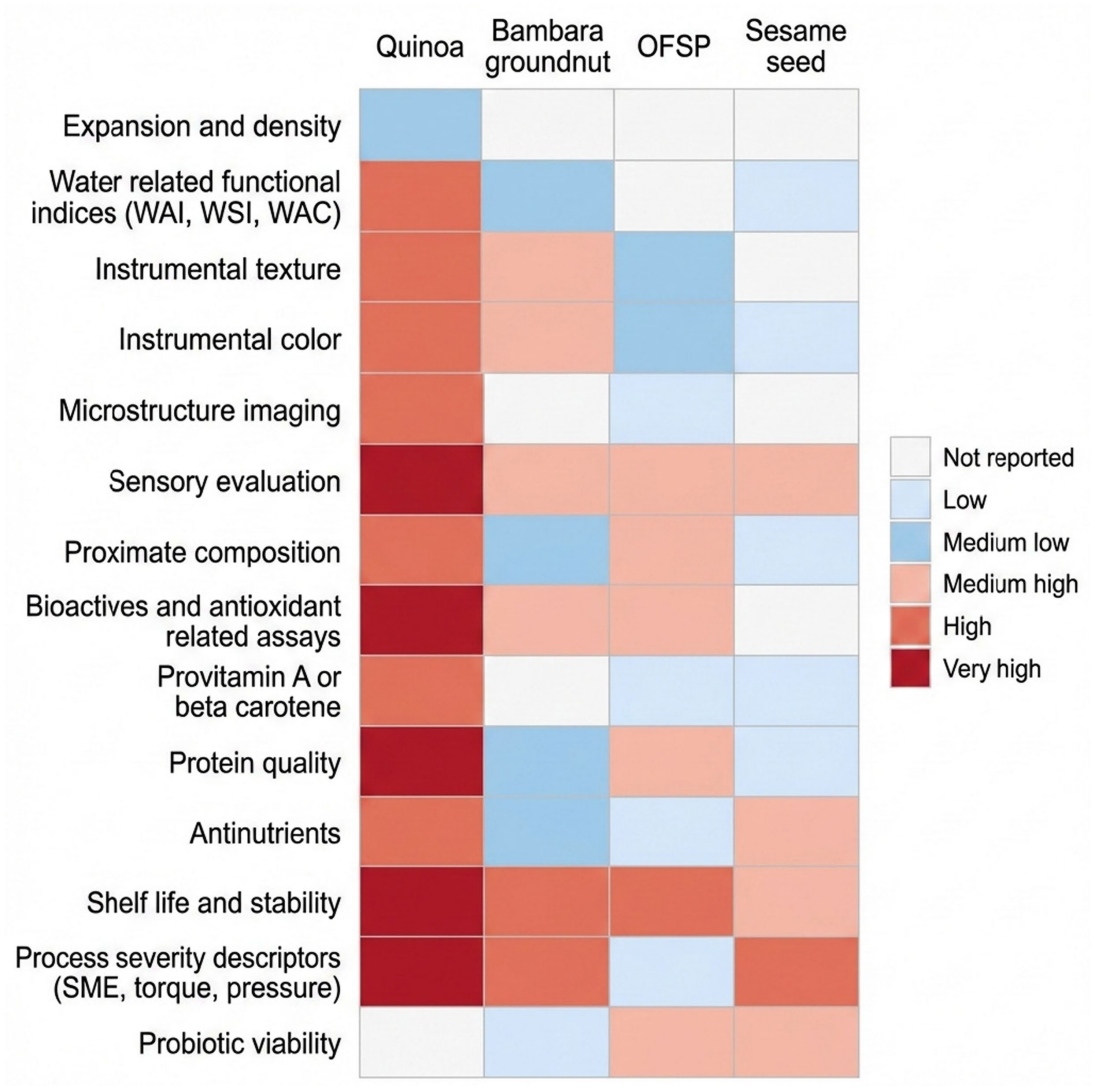
Heatmap-style evidence map showing the relative frequency of outcome categories reported across quinoa, Bambara groundnut, orange-fleshed sweet potato, and sesame seed-based extruded product studies included in this scoping review. Cell shading reflects reporting frequency for each outcome category within the study set and shows that expansion and density, hydration indices such as WAI and WSI, instrumental texture, and color are reported more consistently than microstructure imaging, processing severity descriptors such as SME, torque, and die pressure, antinutritional factors, and shelf-life testing. The map also shows crop specific concentration of outcomes, with provitamin A or *β*-carotene measured mainly in orange-fleshed sweet potato products, oxidation stability assessed mainly in sesame enriched snacks and orange-fleshed sweet potato plus Bambara groundnut formulations, and probiotic viability assessed in quinoa-based second-generation snack studies (15, 23–25, 28, 33–38, 43, 44, 46, 49–58, 60–62). WAI, WSI, WAC, and SME refer to water absorption index, water solubility index, water absorption capacity, and SME, respectively.

Among the included studies, the reported outcomes focused primarily on physical structure and hydration-related endpoints. Expansion indices, bulk or unit density, hardness or puncture force, color parameters, WAI, and WSI were reported much more frequently than sensory properties, shelf-life, and nutritional composition, including proximate composition and provitamin A retention. This emphasis is evident in quinoa extrusion work and comparative whole grain benchmarks, where expansion and density metrics, along with WAI and WSI, were repeatedly used as primary response variables, often alongside porosity estimates or microscopy-based descriptions (34, 36, 43, 44). A similar finding appears in Bambara groundnut studies that applied mixture or response surface designs, where expansion ratio, bulk density, and water indices formed the core basis for optimization and formulation screening (51, 52). In OFSP and sesame seed-enriched snacks, expansion and density were also frequently paired with instrumental color and texture, consistent with the sensitivity of puffing behavior to formulation shifts that increase sugars, protein, or lipids in the melt phase (23, 53, 56). The distribution of reported outcome categories for the four target crops is summarized in Table 6. A visual representation of the same evidence map is provided in Figure 3 to facilitate quick comparison of outcome coverage across the crops.

**Table 6:** Evidence map showing outcome categories reported included studies by crop, quinoa, Bambara groundnut, orange-fleshed sweet potato, and sesame seed.

Quality attribute/outcome	Quinoa	Bambara groundnut	OFSP	Sesame seed
Expansion and density	Low	Not reported	Not reported	Not reported
Water-related functional indices (WAI, WSI, WAC)	High	Medium low	Not reported	Low
Instrumental texture	High	Medium high	Medium low	Not reported
Instrumental color	High	Medium high	Medium low	Low
Microstructure imaging	High	Not reported	Low	Not reported
Sensory evaluation	Very high	Medium high	Medium high	Medium high
Proximate composition	High	Medium low	Medium high	Low
Bioactives and antioxidant capacity	Very high	Medium high	Medium high	Not reported
Provitamin A/β-carotene	High	Not reported	Low	Low
Protein content	Very high	Medium low	Medium high	Low
Anti-nutritional factors	High	Medium low	Low	Medium high
Shelf-life and stability	Very high	High	High	Medium high
Processing severity descriptors (SME, torque, pressure)	Very high	High	Low	High
Probiotic viability	Not reported	Low	Medium high	Medium high

Sensory evaluation and storage stability were reported less consistently than physical indices, although several studies provide strong examples of how these endpoints can support product development decisions. Sensory texture and acceptability were integrated into quinoa and composite snack studies that linked product liking and texture perception to measurable cellular structure, including X-ray microtomography-based pore descriptors, image-based porosity estimates, and fracture-related instrumental metrics (46, 48). Shelf-life assessment was present in selected Bambara groundnut and OFSP snack studies that used peroxide value to track oxidative change and estimate storage stability across formulations, and in millet Bambara groundnut puffed snacks where storage temperature affected microbial quality and sensory scores (23, 28, 50). Probiotic stability across storage was also assessed in quinoa extrudates using *B. coagulans*, providing a rarer example of functional shelf-life testing beyond crispness retention and oxidation indices (25, 46).

Nutritional outcomes were unevenly distributed across crops and were often secondary to structure screening, particularly in quinoa and Bambara groundnut snack studies. OFSP studies most often included provitamin A or *β*-carotene measurements, with reported processing losses and formulation-dependent retention patterns that support the need to treat provitamin A as a quality target rather than an assumed outcome of inclusion (28, 54, 55). Protein quality measurements were reported in a smaller subset of OFSP composite extrudate studies, including *in vitro* protein digestibility and available lysine, and these endpoints were rarely integrated with detailed snack structure metrics in the same paper (15, 58). Antinutritional factors reporting was also limited and tended to appear in studies where ingredient processing such as fermentation, germination, or composite blending was part of the design, rather than in expansion focused snack optimization studies (24, 49, 59).

Reporting of extrusion processing conditions remains a major constraint for cross-study synthesis. Feed moisture, barrel or die temperature, and screw speed were commonly reported, but energy-related descriptors such as SME, torque, and die pressure were included in only a subset of studies, limiting comparability of processing severity across different extruders and formulations (31, 36, 37, 52). In addition, reporting of die geometry, screw configuration, and residence time proxies was inconsistent across the evidence base, which restricts generalization of formulation effects across crop categories and product formats. Table 6 summarizes these patterns and identifies where evidence is concentrated and where outcome reporting remains sparse.

## 4 Discussion

### 4.1 Scope of the evidence and limits for synthesis

Across the four target crops, most extrusion studies aimed to optimize snack structure, resulting in the most robust evidence for expansion indices, density, hydration indices such as WAI and WSI, instrumental texture, and instrumental color. This consistent finding appears repeatedly in quinoa studies across various formulations (34, 36, 37), in Bambara groundnut modeling studies that examine expansion ratio and density alongside water indices (51, 52), and in OFSP and sesame snacks where expansion and density are often reported together with color and texture (23, 53, 54, 56). This focus allows for comparisons of how formulation and processing influence structure formation across crops, but it also limits cross-crop conclusions regarding shelf-life, antinutrients, and nutrient bioavailability, as these outcomes were assessed in a smaller subset of studies (23, 24, 28, 49, 50).

Mechanistic interpretation is most robust when studies report processing severity descriptors that detail the mechanical work applied to the melt. SME (SME), torque, and die pressure were reported in only a subset of the included studies. However, when available, they provide a stronger basis for comparing processing intensity than set points alone, as the same temperature and screw speed can produce different mechanical energy inputs across extruder designs and feed rates (31, 36, 37, 52). Within the scope of this review, studies that combined severity descriptors with expansion and texture outcomes offer the clearest connections between process conditions and final product quality (31, 36, 37, 52).

### 4.2 Process variables and physicochemical transformations that drive snack structure

The reported outcome patterns align with a process-driven sequence in which feed moisture, temperature profile, and shear determine melt plasticization, pressure development at the die, and the balance between starch conversion and molecular fragmentation. In quinoa systems, changes in WSI and WAI were interpreted in relation to extrusion-induced starch transformations, including dextrinization, providing evidence that hydration indices capture changes in the soluble fraction and matrix water binding after extrusion (34, 43). A similar basis is found in studies that report expansion and density alongside WAI and WSI, where changes in hydration behavior coincide with changes in hardness or fracture behavior, indicating that the transformed matrix influences both bubble growth and cell wall setting (34, 36, 37, 43).

Across crop categories, the influence of formulation composition is most apparent when higher protein or lipid ingredients are introduced into starch-rich bases. In quinoa systems, a high inclusion of protein-enriched quinoa fractions reduced expansion and increased hardness in second-generation snacks, while quinoa added to a maize base increased expansion and reduced density under the tested conditions, demonstrating that the same crop can alter structure in different forms depending on ingredient type and matrix composition (33, 46). Comparable structural consequences were reported when sesame ingredients were added to maize, where higher levels of sesame powder or sesame oil cake reduced expansion and increased puncture force, with SEM observations showing altered cell morphology consistent with denser textures (23, 56). Bambara groundnut modeling studies similarly report expansion ratio and density as functions of Bambara groundnut content, moisture, and screw speed, supporting the conclusion that composite matrices require co-optimization of formulation and processing rather than fixed process conditions across blends (51, 52).

### 4.3 Quinoa based extrudates, structure performance, and functional design

Quinoa has the most comprehensive evidence base in this review and includes a broader range of product concepts than the other crops, from directly expanded cereal blends to quinoa-rich matrices, sprouted ingredients, and probiotic snacks (33, 35, 38, 43, 46). Comparisons across studies show that quinoa does not exert a uniform effect on expansion. In maize-based extrudates, the addition of quinoa was associated with reduced bulk density and increased expansion and porosity, with microstructural observations consistent with enhanced expansion (33). In contrast, a high inclusion of high-protein quinoa flour in second-generation snacks reduced expansion and increased hardness, indicating that the functional contribution of quinoa depends on the fraction type and inclusion level, not merely on the presence of quinoa (46). Protein-enriched pseudocereal extrudates, along with microstructural and sensory evaluations, provide additional support that increasing protein content can influence structural and textural behavior relative to cereal amounts, although direct alignment of processing strategies across studies remains limited (32, 36).

The strongest evidence linking structure to perception in the quinoa group comes from studies that quantified pore structure and paired it with sensory texture. In maize-based extrudates containing quinoa, perceived crispness, crunchiness, hardness, and adhesiveness were associated with pore structure metrics quantified by X-ray microtomography, indicating that cellular architecture provides a mechanistic bridge between expansion indices and sensory responses (48). When image-based porosity or fracture metrics were paired with consumer acceptability in probiotic snacks, the interpretation of texture and liking became more robust than reliance on expansion alone (46).

Quinoa studies also illustrate how ingredient pretreatment alters post-extrusion functionality. Sprout-based formulations altered hydration behavior and bioactive compounds in extrudates, supporting the view that germination modifies the balance between soluble fragments and structure forming components that remain after extrusion (35, 38). Functional concepts extend further in probiotic fortified quinoa snacks where *Bacillus coagulans* viability during storage and resistance under simulated gastrointestinal conditions were quantified, indicating that the snack matrix can be engineered for delivery outcomes beyond texture and appearance (40, 46). However, this evidence remains limited to a small number of studies (citations), so generalizable design rules for probiotic survival across extrusion conditions are not yet supported by the available literature.

### 4.4 Bambara groundnut extrudates, protein enrichment, and formulation constraints

Bambara groundnut studies emphasize protein enrichment and formulation screening through response surface and mixture designs, using expansion ratio, bulk density, WAI, WSI, and texture metrics such as breaking force as primary outcomes (51, 52). A consistent observation is that structural outcomes depend jointly on Bambara groundnut addition level and process variables, particularly moisture and screw speed, supporting a co-optimization framework where higher Bambara groundnut inclusion often coincides with reduced expansion under the tested conditions (51, 52). This agreement across modeling studies enhances confidence that Bambara groundnut -driven enrichment is likely to introduce expansion challenges unless the starch base and processing strategy are adjusted accordingly (51, 52).

A distinct line of evidence differentiates blend ratio effects from ingredient pretreatment effects. In OFSP and Bambara groundnut co-extruded snacks, increasing the Bambara groundnut proportion increased protein, fat, crude fiber, ash, and energy content, whereas increasing OFSP raised provitamin A levels, indicating that blend ratios can be optimized for either protein density or provitamin A delivery depending on product goals (28). In a separate formulation where ingredient ratios were fixed and Bambara groundnut seeds were pretreated before milling, roasting and germination reduced antinutrient activities and altered functional properties, with the most significant reduction in trypsin inhibitor activity observed when germination was followed by roasting. Roasted and germinated flours exhibited higher protein content than untreated and germinated flours, and roasted Bambara groundnut flour achieved the highest sensory acceptability for taste and aroma-related attributes (49). These results suggest that pretreatments can mitigate antinutrient concerns and improve acceptability without altering blend ratios, which is relevant when expansion issues affect Bambara groundnut inclusion in puffed products (49, 51, 52).

Storage stability has been addressed more directly in Bambara groundnut containing products than in several quinoa and OFSP snack studies. Microbial quality and sensory changes were monitored over storage time and temperature in pearl millet and Bambara groundnut extrudates, showing that storage conditions can drive quality loss even when initial structure is acceptable (50). In OFSP and Bambara groundnut snacks, peroxide value-based modeling indicated a shorter predicted shelf-life at higher Bambara groundnut proportions, consistent with greater oxidation risk in higher lipid formulations within that product category (28).

### 4.5 OFSP extrudates, provitamin a retention, and structure challenges

OFSP studies are distinguished by routine measurement of provitamin A or *β*-carotene-related endpoints alongside expansion, density, color, texture, and acceptability outcomes (15, 28, 53, 54). This choice of outcomes aligns with OFSP’s positioning as a provitamin A ingredient, but the data show that retention depends on both the matrix and the severity of processing. In OFSP and Bambara groundnut snacks, provitamin A losses were reported and varied with blend ratios and conditions, indicating that inclusion alone is insufficient and retention must be treated as an optimization target (28). Greater emphasis on temperature is necessary because the OFSP studies that reported extrusion conditions operated within temperature ranges where carotenoids are measurably lost during extrusion, and the extent of retention varied across the tested temperature settings and formulations. Among the studies that reported operating conditions, puffed snack systems typically used barrel or die temperatures between 100 °C and 130 °C, including designs that compared 100 °C and 120 °C. Some multi-zone profiles in nutrition-focused extrudates even reached 145 °C in the barrel (28, 53, 59). Within these temperature ranges, losses of provitamin A were observed in OFSP-based extrudates, with retention differing across blend ratios and operating conditions, including temperature and screw speed (28). Losses were also reported in extruded maize and OFSP pasta, highlighting the broader sensitivity of carotenoids to thermal and oxidative exposure during extrusion, even when the product format differs from directly expanded snacks (55). Because complete temperature profiles, residence time proxies, and severity descriptors are inconsistently reported in OFSP studies, the existing literature does not support a single temperature threshold for provitamin A degradation that can be generalized across formulations and extruder configurations.

The OFSP literature also shows that micronutrient targets interact with appearance and acceptability. In sorghum-based snacks, increasing OFSP levels resulted in higher vitamin A-related values and produced darker, more reddish products, while protein and fiber content decreased, and overall acceptability remained highest for formulations with a higher sorghum fraction within that system (54). Process optimization studies further indicate that moisture and extrusion temperature interact with formulation to shape expansion ratio, porosity, and density, which are key texture determinants in puffed snacks, and higher temperature settings can improve porosity and acceptability within the tested parameters (53). These observations support a practical interpretation in which targeting provitamin A needs to be balanced against structural development and sensory drivers, rather than treated as a simple function of OFSP inclusion (53, 54).

Multi-ingredient OFSP formulations also show that micronutrient goals can be combined with broader nutrient density targets. Mixture design snacks combining beans, maize, amaranth, and OFSP reported improved nutrient density and *in vitro* protein digestibility with adult and child acceptability testing, showing that OFSP can be used in composite strategies where both macro and micronutrients are considered (15). Protein quality attributes were also addressed in a smaller set of OFSP composite studies through *in vitro* protein digestibility and available lysine, providing a way to evaluate nutritional quality beyond proximate composition in systems that pair OFSP with protein-rich ingredients (15, 58). Complementary food-style extrudates report antinutrient and mineral outcomes after extrusion and support the broader relevance of measuring these parameters when OFSP is used for nutrition-sensitive formulations, although caution should be exercised in transferring insights to snack texture interpretation due to format differences (59, 60).

### 4.6 Sesame based extrudates, bioactive compounds, oxidation stability, and expansion consequences

Sesame studies align more closely with questions of lipid and oxidative stability than those of other crops. Sesame powder increased phenolics, sesame lignans, and gamma tocopherol, along with sesamin and sesamolin in puffed corn snacks, while the expansion ratio decreased with higher sesame inclusion (23). This combination supports sesame as both a nutritionally and stability-relevant ingredient, but also highlights a consistent structural limitation that must be managed in puffed products (23). Similar expansion penalties and texture increases were reported when semi-defatted sesame oil cake was incorporated into maize expanded extrudates, with reduced sectional expansion index, higher puncture force, and SEM evidence of modified cell morphology (56).

Sesame is also featured in studies where ingredient processing is part of the design. In cassava-based extruded snacks followed by frying, fermented and germinated sesame flours improved proximate composition and reduced antinutrient levels compared to flour blends, and higher inclusion of fermented sesame achieved high sensory acceptability within that product category (24). A separate study on extruded sesame byproduct beverages reported changes in phytochemical profiles and *in vitro* bioactivity, supporting the broader view that extrusion can preserve or transform sesame-related phytochemicals; however, the translation of those findings to expanded snack structure is not established by that study design (57).

### 4.7 Agreements, discrepancies, and implications for future study design

Two agreements are consistent across crops within the screened and included literature. First, structure outcomes such as expansion and density are highly sensitive to the interaction of formulation with moisture and thermal conditions, and cross-study comparability improves when processing severity descriptors are reported (31, 36, 37, 52). Second, higher inclusion of protein-rich or lipid-rich ingredients commonly coincides with reduced expansion and increased hardness-related metrics at higher inclusion levels, reported for high quinoa fractions in second-generation snacks, Bambara groundnut in composite designs, and sesame in maize extrudates (23, 46, 51, 52, 56).

The main discrepancies are informative rather than problematic because they point to matrix dependence. Quinoa improved expansion in a maize-based product under one processing method, yet reduced expansion at high inclusion in other systems, which is consistent with differences in starch to protein balance and ingredient form across studies (32, 33, 46). OFSP studies agree on the need to measure provitamin A and *β*-carotene, yet reported losses show that retention depends on processing and matrix, supporting retention as a response variable rather than an assumed benefit (28, 53–55).

Several reporting gaps limit transferability of conclusions. Energy descriptors such as SME, torque, and die pressure remain inconsistent across studies, restricting comparison of processing severity across extruders (31, 36, 37, 52). Microstructure tools such as SEM, X-ray microtomography, and image-based porosity were applied effectively in selected quinoa and sesame studies and strengthened links between structure and sensory texture, but similar approaches were less frequent in Bambara groundnut and OFSP snack studies within the reviewed body of work (33, 46, 48, 56). Shelf-life testing was based in studies focused on oxidation, microbial stability, or probiotic viability, leaving limited direct evidence on humidity-driven crispness loss during storage, despite its relevance to extruded snack acceptability (23, 25, 28, 40, 46, 50).

Within the scope of this review, the included studies support the feasibility of quinoa, Bambara groundnut, OFSP, and sesame seed for extruded snack development and identify formulation challenges that can inform ingredient selection and process design. More generalizable conclusions will depend on more consistent reporting of processing severity descriptors and on routine integration of microstructure and sensory measurements with expansion, density, and hydration indices, particularly for Bambara groundnut and OFSP systems where nutritional objectives are prominent but texture mechanisms are less frequently quantified (48, 51–54).

## 5 Practical implications for formulation and process design and future studies

Evidence across the four target crops supports formulation and processing choices that expansion and texture should be considered as design outcomes rather than fixed properties of the ingredient. In quinoa-based systems, both positive and negative effects on expansion have been reported depending on base matrix and inclusion level. Quinoa addition increased expansion and reduced bulk density in maize-based extrudates under the tested conditions, whereas high inclusion of high-protein quinoa flour in rice-based second-generation snacks reduced expansion and increased hardness, indicating that the balance between starch-driven puffing and protein rich fractions is a primary formulation constraint (33, 46). Similar tradeoffs appear in complex pseudocereal extrudates with added protein, where reduced expansion and higher hardness were observed relative to commercial cereal benchmarks, supporting cautious interpretation when protein enrichment is combined with high fiber pseudocereal matrices (32). For Bambara groundnut, response surface and mixture design studies confirm that expansion ratio and bulk density depend jointly on Bambara groundnut level, feed moisture, and screw speed, supporting a formulation approach in which Bambara groundnut addition is paired with sufficient starch rich components and a process window that preserves bubble growth and setting (51, 52). Sesame seed ingredients behave similarly, where sesame seed powder or semi defatted cake reduced expansion and increased hardness-related metrics in maize-based extrudates as inclusion increased, consistent with lipid and protein effects on puffing in expanded snacks (23, 56). These findings suggest that high inclusion levels of protein-rich or lipid-rich underutilized crop ingredients are more likely to succeed when the base matrix retains adequate starch expansion capacity, when particle size and ingredient form are selected to support melt continuity, and when the extrusion window is adjusted to manage viscosity and die expansion rather than held constant across formulations (31, 36).

Processing variables that control water plasticization and thermal history appear repeatedly as dominant drivers of product structure, which supports practical emphasis on the feed moisture and temperature profile during formulation screening. In quinoa extrudates, feed moisture and temperature influenced expansion, density, hardness, WAI, and WSI, and studies reporting energy descriptors such as SME, torque, or die pressure provide a stronger basis for comparing process intensity across conditions (31, 36). Similar modeling approaches in millet and Bambara groundnut extrudates demonstrate that SME and residence time can be considered as outputs of formulation and process set points, supporting the inclusion of energy descriptors in future snack studies to improve cross-study comparability (52). For OFSP-enriched snacks, optimization studies confirm that moisture and extrusion temperature shape expansion ratio, porosity, and density, indicating that process screening should include these variables when color development and provitamin A retention are also targeted (53).

Crop specific quality targets provide additional practical constraints. For OFSP provitamin A-related endpoints should be treated as response variables that must be quantified and optimized alongside expansion and sensory acceptance. Provitamin A losses were reported in OFSP and Bambara groundnut coextruded snacks, and *β*-carotene losses were also observed in maize-OFSP extruded pasta, indicating sensitivity to processing severity and matrix effects (28, 55). Studies that report carotenoid content together with color and acceptability data show that OFSP inclusion can increase vitamin A-related measures and shift color, but acceptability remains formulation dependent, supporting the need to balance nutritional targets with sensory drivers in snack design (15, 54). For sesame seed, storage stability and rancidity risk are central considerations because sesame seed enrichment changes lipid composition and introduces an oxidation management problem rather than only a structure problem. Peroxide value and acidity value were tracked during storage in sesame seed fortified maize snacks, supporting the need for shelf-life testing when sesame seed is used as a functional ingredient in expanded products (23). OFSP and Bambara groundnut snacks also used peroxide value-based modeling, and storage trials of millet and Bambara groundnut puffed snacks showed temperature driven microbial and sensory deterioration, supporting the importance of packaging and storage conditions for enriched extrudates (28, 50). For quinoa, functional snack concepts such as probiotic delivery introduce additional design choices regarding probiotic incorporation route. Both pre-extrusion inclusion and post-extrusion surface application were evaluated for *B. coagulans*, with storage viability and simulated gastrointestinal resistance quantified, supporting feasibility while also indicating that processing severity and post-processing conditions are critical for maintaining viable counts (40, 46).

Future studies would benefit from more consistent reporting and a tighter alignment between mechanistic measurements and consumer relevant outcomes. Energy descriptors such as SME, torque, and die pressure should be reported more consistently, since studies that include these parameters enable stronger comparison of extrusion severity across crops, machines, and formulations (31, 36, 52). Microstructure analysis were used effectively in quinoa and sesame seed studies to connect pore architecture and cell morphology to texture and sensory properties, and wider adoption of these approaches would strengthen the ability to generalize structure texture relationships across underutilized crop systems (33, 46, 48, 56). For OFSP, provitamin A retention should be quantified using validated methods and interpreted together with color development and acceptability, because inclusion alone does not ensure retention and losses have been documented across product formats (28, 55). Protein quality endpoints such as *in vitro* protein digestion and available lysine can add nutritional meaning beyond proximate composition, yet these measures were reported in only a subset of OFSP composite extrudate studies, suggesting that wider integration of protein quality metrics is needed, particularly when legume and pseudocereal enrichment is a primary objective (15, 58). Antinutrient reporting remains limited and appears mainly in studies that explicitly targeted fermentation, germination, or ingredient preprocessing, indicating that future snack studies that use these approaches should consistently quantify antinutritional factors alongside sensory and structure outcomes to support both nutritional and safety-related conclusions (24, 49, 59). Finally, shelf-life research remains sparse across most crop categories beyond lipid oxidation and probiotic viability, and texture retention under humidity exposure is rarely quantified in the literature, despite its direct relevance to consumer acceptance and commercial stability (23, 28, 40, 46).

Future evidence mapping should extend this framework to additional nutrient dense underutilized crops, including African yam bean and fonio, to test whether the same formulation tradeoffs and reporting gaps persist across a wider crop set.

## 6 Conclusion

Evidence on underutilized crops in extruded snacks is expanding but remains uneven across crops and outcome categories. Quinoa has the most extensively developed evidence-base and is consistently evaluated using expansion indices, density, WAI, WSI, and instrumental texture. In addition, several studies extend outcome assessment to microstructural characteristics, probiotic viability, and digestibility-related parameters. Bambara groundnut studies primarily support protein enrichment within composite snacks and show formulation and moisture sensitivity for expansion and density, with limited but informative storage quality assessments. OFSP-based research consistently measures provitamin A or *β*-carotene and reports that retention is not guaranteed, supporting the need to optimize processing conditions alongside sensory and color targets. Sesame seed studies demonstrate clear enrichment in phenolics, lignans, and tocopherols and provide stronger coverage of oxidation-related shelf-life indicators, while reporting expansion penalties at higher inclusion. Across all crops, improved comparability and translation would benefit from more consistent reporting of extrusion severity descriptors and a broader inclusion of sensory, shelf-life, protein quality, and antinutrient endpoints matched to the intended product function. Future scoping reviews should extend the crop scope to additional underutilized crops to improve generalizability.

## References

[1] Alam MS Kaur J Khaira H Gupta K. Extrusion and extruded products: changes in quality attributes as affected by extrusion process parameters: a review. Crit Rev Food Sci Nutr. (2016) 56:445–73. doi: 10.1080/10408398.2013.779568, 25574813

[2] Ali IM Forsido SF Kuyu CG Ahmed EH Andersa KN Chane KT . Effects of extrusion process conditions on nutritional, anti-nutritional, physical, functional, and sensory properties of extruded snack: a review. Food Sci Nutr. (2024) 12:8755–61. doi: 10.1002/fsn3.4472, 39620018 PMC11606806

[3] Grasso S. Extruded snacks from industrial by-products: a review. Trends Food Sci Technol. (2020) 99:284–94. doi: 10.1016/j.tifs.2020.03.012

[4] Shah FUH Sharif MK Butt MS Shahid M. Development of protein, dietary fiber, and micronutrient enriched extruded corn snacks. J Texture Stud. (2017) 48:221–30. doi: 10.1111/jtxs.12231, 28573729

[5] Korkerd S Wanlapa S Puttanlek C Uttapap D Rungsardthong V. Expansion and functional properties of extruded snacks enriched with nutrition sources from food processing by-products. J Food Sci Technol. (2016) 53:561–70. doi: 10.1007/s13197-015-2039-1, 26787975 PMC4711464

[6] Tyl C Bresciani A Marti A. Recent progress on improving the quality of bran-enriched extruded snacks. Foods. (2021) 10:2024. doi: 10.3390/foods10092024, 34574134 PMC8471519

[7] Leonard W Zhang P Ying D Fang Z. Application of extrusion technology in plant food processing byproducts: an overview. Compr Rev Food Sci Food Saf. (2020) 19:218–46. doi: 10.1111/1541-4337.12514, 33319515

[8] Huang X Liu H Ma Y Mai S Li C. Effects of extrusion on starch molecular degradation, order–disorder structural transition and digestibility—a review. Foods. (2022) 11:2538. doi: 10.3390/foods11162538, 36010538 PMC9407177

[9] Esen BO Bozdogan N Kutlar LM Kumcuoglu S. Development of gluten-free extruded snack containing lentil flour and evaluation of extrusion process conditions on quality properties. Food Sci Nutr. (2025) 13:e70663–20. doi: 10.1002/fsn3.70663, 40735399 PMC12301571

[10] G. V. Research, “Snacks market size, share & trends analysis report by product type, by distribution channel, by region, and segment forecasts, 2025–2030,” Grand View Research. (2024). Available online at: https://www.grandviewresearch.com/industry-analysis/snacks-market

[11] G. V. Research, “Extruded snacks market size, share & trends analysis report by product type, by distribution channel, by region, and segment forecasts, 2025–2030,” Grand View Research. (2025). Available online at: https://www.grandviewresearch.com/industry-analysis/extruded-snacks-market

[12] Alefew YD Tiruneh AT Yehuala TF. Optimization of extrusion conditions for development of high quality rice-lupin-pumpkin based extruded snack food. Heliyon. (2024) 10:e40913. doi: 10.1016/j.heliyon.2024.e40913, 39720086 PMC11667639

[13] Gomes KS Berwian GF Batistella VMC Bender LE Reinehr CO Colla LM. Nutritional and technological aspects of the production of proteic extruded snacks added of novel raw materials. Food Bioprocess Technol. (2023) 16:247–67. doi: 10.1007/s11947-022-02887-0

[14] Marincas M Marc R Muresan C. Overview of expanded and extruded products. Bull Univ Agric Sci Vet Med Cluj-Napoca Food Sci Technol. (2025) 82:31–43. doi: 10.15835/buasvmcn-fst:2024.0034

[15] Natabirwa H Nakimbugwe D Lung'aho M Tumwesigye KS Muyonga JH. Bean-based nutrient-enriched puffed snacks: formulation design, functional evaluation, and optimization. Food Sci Nutr. (2020) 8:4763–72. doi: 10.1002/fsn3.1727, 32994938 PMC7500791

[16] Wani SA Ganie NA Kumar P. Quality characteristics, fatty acid profile and glycemic index of extrusion processed snacks enriched with the multicomponent mixture of cereals and legumes. Legume Sci. (2021) 3:3–9. doi: 10.1002/leg3.76

[17] Toledo VCS Carvalho CWP Vargas-Solórzano JW Ascheri JLR Comettant-Rabanal R. Extrusion cooking of gluten-free whole grain flour blends. J Food Process Eng. (2020) 43:1–9. doi: 10.1111/jfpe.13303

[18] Oliveira DPL Soares Júnior MS Bento JAC dos Santos IG de Ferreira TAP. Influence of extrusion conditions on the physical and nutritional properties of snacks from maize and pearl millet. J Food Process Preserv. (2021) 45:1–9. doi: 10.1111/jfpp.15215

[19] Ali A Bhattacharjee B. Nutrition security, constraints, and agro-diversification strategies of neglected and underutilized crops to fight global hidden hunger. Front Nutr. (2023) 10:1144439. doi: 10.3389/fnut.2023.1144439, 37426189 PMC10324569

[20] Balakrishnan G Schneider RG. The role of Amaranth, quinoa, and millets for the development of healthy, sustainable food products—a concise review. Foods. (2022) 11:2442. doi: 10.3390/foods11162442, 36010444 PMC9407507

[21] Chelangat M Muturi P Gichimu B Gitari J Mukono S. Nutritional and phytochemical composition of Bambara groundnut (*Vigna subterranea* [L.] Verdc) landraces in Kenya. Int J Agron. (2023) 2023:1–11. doi: 10.1155/2023/9881028

[22] Alomatu DE Yankey S Gyimah L Eshun G. Nutritional profile and functional properties of Orange-fleshed sweet potato, Bambara groundnut, and Brown Rice blended complementary food. Int J Food Sci. (2025) 2025:2025. doi: 10.1155/ijfo/5397972, 41098858 PMC12519420

[23] Hashempour-Baltork F Torbati M Azadmard-Damirchi S Savage GP. Quality properties of puffed corn snacks incorporated with sesame seed powder. Food Sci Nutr. (2018) 6:85–93. doi: 10.1002/fsn3.532, 29387365 PMC5778234

[24] Olorode OO Sobowale SS. Evaluation of qualities of extruded snacks from yellow cassava flour substituted with processed sesame seed’s flour. World J Adv Res Rev. (2021) 10:074–86. doi: 10.30574/wjarr.2021.10.1.0134

[25] Muñoz-Pabon KS Roa-Acosta DF Hoyos-Concha JL Bravo-Gómez JE Ortiz-Gómez V. Quinoa snack production at an industrial level: effect of extrusion and baking on digestibility, bioactive, rheological, and physical properties. Foods. (2022) 11:3383. doi: 10.3390/foods11213383, 36359997 PMC9658072

[26] Kuktaite R Repo-Carrasco-Valencia R de Mendoza CCH Plivelic TS Hall S Johansson E. Innovatively processed quinoa (*Chenopodium quinoa* Willd.) food: chemistry, structure and end-use characteristics. J Sci Food Agric. (2022) 102:5065–76. doi: 10.1002/jsfa.11214, 33709442

[27] Faliarizao N Berrios JDJ Dolan KD. Value-added processing of food legumes using extrusion technology: a review. Legum Sci. (2024) 6:1–16. doi: 10.1002/leg3.231

[28] Honi B Mukisa IM Mongi RJ. Proximate composition, provitamin a retention, and shelf life of extruded orange-fleshed sweet potato and Bambara groundnut-based snacks. J Food Process Preserv. (2018) 42:1–8. doi: 10.1111/jfpp.13415

[29] Okafor CA Falade KO. Nutritional, physicochemical, and sensory characteristics of extruded Bambara groundnut (*Vigna subterranea*)-based ready-to-eat breakfast cereal. J Food Process Preserv. (2021) 45:1–11. doi: 10.1111/jfpp.15347

[30] Pham MT Rajić A Greig JD Sargeant JM Papadopoulos A Mcewen SA. A scoping review of scoping reviews: advancing the approach and enhancing the consistency. Res Synth Methods. (2014) 5:371–85. doi: 10.1002/jrsm.1123, 26052958 PMC4491356

[31] Ramos Diaz JM Sundarrajan L Kariluoto S Lampi AM Tenitz S Jouppila K. Effect of extrusion cooking on physical properties and chemical composition of corn-based snacks containing amaranth and quinoa: application of partial least squares regression. J Food Process Eng. (2017) 40:e12320. doi: 10.1111/jfpe.12320

[32] Leiva-Castro B Mamani-Benavente L Elías-Peñafiel C Comettant-Rabanal R Silva-Paz R Olivera-Montenegro L . Andean Pseudocereal flakes with added pea protein isolate and Banana flour: evaluation of physical-chemical, microstructural, and sensory properties. Foods. (2025) 14:1–18. doi: 10.3390/foods14040620, 40002068 PMC11854138

[33] Cueto M Porras-Saavedra J Farroni A Alamilla-Beltrán L Schöenlechner R Schleining G . Physical and mechanical properties of maize extrudates as affected by the addition of chia and quinoa seeds and antioxidants. J Food Eng. (2015) 167:139–46. doi: 10.1016/j.jfoodeng.2015.07.027

[34] Mindani C Baldeón EO Ibáñez V Calizaya F Taipe C Zegarra J . Effects of moisture content and lime concentrate on physiochemical, mechanical, and sensory properties of quinoa snacks: an ancient Andean crop in Puno, Peru. AgriEngineering. (2024) 6:3931–51. doi: 10.3390/agriengineering6040223

[35] Afsharfar M BeigMohammadi Z Milani E Jafari SM. Optimizing nutritional and functional properties of gluten-free grass pea and quinoa instant flours: dual modification by sprouting and extrusion. Food Sci Nutr. (2025) 13:e70697. doi: 10.1002/fsn3.70697, 40831945 PMC12359068

[36] Robin FF Théoduloz C Srichuwong S Theoduloz C Srichuwong S. Properties of extruded whole grain cereals and pseudocereals flours. Int J Food Sci Technol. (2015) 50:2152–9. doi: 10.1111/ijfs.12893

[37] Dogan H Karwe MV Doğan H Karwe MV. Physicochemical properties of quinoa extrudates. Food Sci Technol Int. (2003) 9:101–14. doi: 10.1177/108201303033940

[38] Paucar-Menacho LM Schmiele M Lavado-Cruz AA Verona-Ruiz AL Mollá C Peñas E . Andean sprouted pseudocereals to produce healthier extrudates: impact in nutritional and physicochemical properties. Foods. (2022) 11:3259. doi: 10.3390/foods11203259, 37431004 PMC9601839

[39] Torres Vargas OL Lema González M Galeano Loaiza YV. Optimization study of pasta extruded with quinoa flour (*Chenopodium quinoa* willd). CYTA - J Food. (2021) 19:220–7. doi: 10.1080/19476337.2021.1883116

[40] Pabon KSM Concha JLH Duque JFS Muñoz Pabon KS Hoyos Concha JL Solanilla Duque JF. Quinoa extruded snacks with probiotics: physicochemical and sensory properties. Front Sustain Food Syst. (2022) 6:935425. doi: 10.3389/fsufs.2022.935425

[41] Córdoba-Cerón DM Bravo-Gómez JE Agudelo-Laverde LM Roa-Acosta DF Nieto-Calvache JE. Techno-functional properties of gluten-free pasta from hyperprotein quinoa flour. Heliyon. (2023) 9:e18539. doi: 10.1016/j.heliyon.2023.e18539, 37560662 PMC10407035

[42] Paucar-Menacho LM Schmiele M Vásquez Guzmán JC Rodrigues SM Simpalo-Lopez WD . Smart pasta design: tailoring formulations for technological excellence with sprouted quinoa and Kiwicha grains. Foods. (2024) 13:353. doi: 10.3390/foods13020353, 38275720 PMC10815487

[43] Taverna LG Leonel M Mischan MM Taverna MM. Changes in physical properties of extruded sour cassava starch and quinoa flour blend snacks. Food Sci Technol. (2012) 32:826–34. doi: 10.1590/S0101-20612012005000113

[44] Alajil O Hymavathi TV Robert P Deepika LVSB. Effect of flour composition and temperature on physico-chemical and sensory properties of quinoa based extrudates. J Pharm Res Int. (2018) 24:1–13. doi: 10.9734/jpri/2018/45346

[45] Gearhart C Rosentrater KA. Extrusion processing of Amaranth and Quinoa into gluten-free snack foods for celiac and gluten-free diets. J Food Res. (2017) 6:107. doi: 10.5539/jfr.v6n5p107

[46] Muñoz Pabon KS Roa Acosta DF Bravo JE. Second-generation snacks prepared from quinoa with probiotic. Physicochemical properties, in vitro digestibility, antioxidant activity and consumer acceptability. Heliyon. (2024) 10:e36525. doi: 10.1016/j.heliyon.2024.e36525, 39258187 PMC11385775

[47] Filipović J Djalovic I Košutić M Nićetin M Lončar B Radosavljević M . Modeling and optimization of extruded corn product fortification. Foods. (2026) 15:208. doi: 10.3390/foods15020208, 41596807 PMC12840189

[48] Ramos Diaz JM Suuronen JP Deegan KC Serimaa R Tuorila H Jouppila K. Physical and sensory characteristics of corn-based extruded snacks containing Amaranth, Quinoa and Kañiwa flour. Lwt. (2015) 64:1047–56. doi: 10.1016/j.lwt.2015.07.011

[49] Okafor JNC Ani JC Okafor GI. Effect of processing methods on qualities of bambara groundnut (*Voandzeia subterranea* (L.) Thouars) flour and their acceptability in extruded snacks (2014). doi: 10.3923/ajft.2014.350.359,

[50] Ajala O Adelusi OA Kajihausa OE Onabanjo OO Oyewole OB Obadina AO. Effect of storage temperature and time on the microbial quality and sensory properties of extrudates produced from pearl millet (*Pennisetum glaucum* (L) Leake) and Bambara groundnut (*Vigna subterranea*) flour blends. J Stored Prod Res. (2024) 109:102442. doi: 10.1016/j.jspr.2024.102442

[51] Ogunmuyiwa OH Adebowale AA Sobukola OP Onabanjo OO Obadina AO Adegunwa MO . Production and quality evaluation of extruded snack from blends of bambara groundnut flour, cassava starch, and corn bran flour. J Food Process Preserv. (2017) 41:1–7. doi: 10.1111/jfpp.13183

[52] Filli KB Nkama I Jideani VA. The effect of extrusion conditions on the physical and functional properties of millet – Bambara groundnut based Fura. Am J Food Sci Technol. (2013) 1:87–101. doi: 10.12691/ajfst-1-4-5

[53] Sule S Okafor GI Okoyeuzu CF Onah GE. Optimization of process variables for extruded snacks from acha-peanut blends separately enriched with carrot and orange-fleshed sweet potato flours. Turkish J Agric - Food Sci Technol. (2025) 13:2561–9. doi: 10.24925/turjaf.v13i9.2561-2569.7662

[54] Jenfa MD Adelusi OA Aderinoye A Coker OJ Martins IE Oyewole OB . Evaluation of the physicochemical, nutritional, textural, and sensory characteristics of extrudates from sorghum and orange-fleshed sweet potato flour blends. J Food Process Preserv. (2024) 2024:2930130. doi: 10.1155/2024/2930130

[55] Baah RO Duodu KG Emmambux MN. Cooking quality, nutritional and antioxidant properties of gluten-free maize – orange-fleshed sweet potato pasta produced by extrusion. Lwt. (2022) 162:113415. doi: 10.1016/j.lwt.2022.113415

[56] Carvalho CWP Takeiti CY Freitas DDGC Ascheri JLR. Use of sesame oil cake (*Sesamum indicum* L.) on corn expanded extrudates. Food Res Int. (2012) 45:434–43. doi: 10.1016/j.foodres.2011.11.009

[57] Quintero-Soto MF Espinoza-Moreno RJ Félix-Medina JV Salas-López F López-Carrera CF Argüelles-López OD . Comparison of phytochemical profile and in vitro bioactivity of beverages based on the unprocessed and extruded sesame (*Sesamum indicum* L.) seed byproduct. Foods. (2022) 11:3175. doi: 10.3390/foods11203175, 37430924 PMC9601822

[58] Nkesiga J Anyango J Ngoda P. Protein quality of extruded ready-to-eat baby foods from orange-fleshed sweet potato, amaranth seeds, and soybean flour blends. Am J Food Sci Nutr. (2022) 4:24–36. doi: 10.47672/ajfsn.1287

[59] Araro T Gemechu F Wotango A Esho T. Chemical formulation and characterization of complementary foods from blend of orange-fleshed sweet potato, Brown Teff, and dark red kidney beans. Int J Food Sci. (2020) 2020:1–13. doi: 10.1155/2020/4803839, 32509844 PMC7244972

[60] Tenagashaw MW Kenji GM Melaku ET Huyskens-Keil S Kinyuru JN. Proximate composition and selected functional properties of complementary foods from teff fortified with soybean and orange-fleshed sweetpotato. Ruforum Work Doc Ser. (2016) 6:112–965. doi: 10.5539/jfr.v6n1p112

[61] Duru GN Kajihausa OE Olatunde GO Onabanjo OO. Optimization of composite flour for the production of thermo extrudates made from African breadfruit seeds, orange-fleshed sweet potato and tigernut. J Food Sci Technol. (2024) 63:51–64. doi: 10.1007/s13197-024-06114-w, 41684473 PMC12891322

[62] Ramos Diaz J. M. Diaz J. M. R., Use of Amaranth, Quinoa, Kañiwa and Lupine for the Development of Gluten-Free Extruded Snacks. (2015).

[63] Adeola AA Bamgbose OO Ohizua ER. Proximate and functional properties of orange-fleshed sweet potato/pigeon pea flour blends and extrudates. Appl Trop Agric. (2019) 24:1–12.

